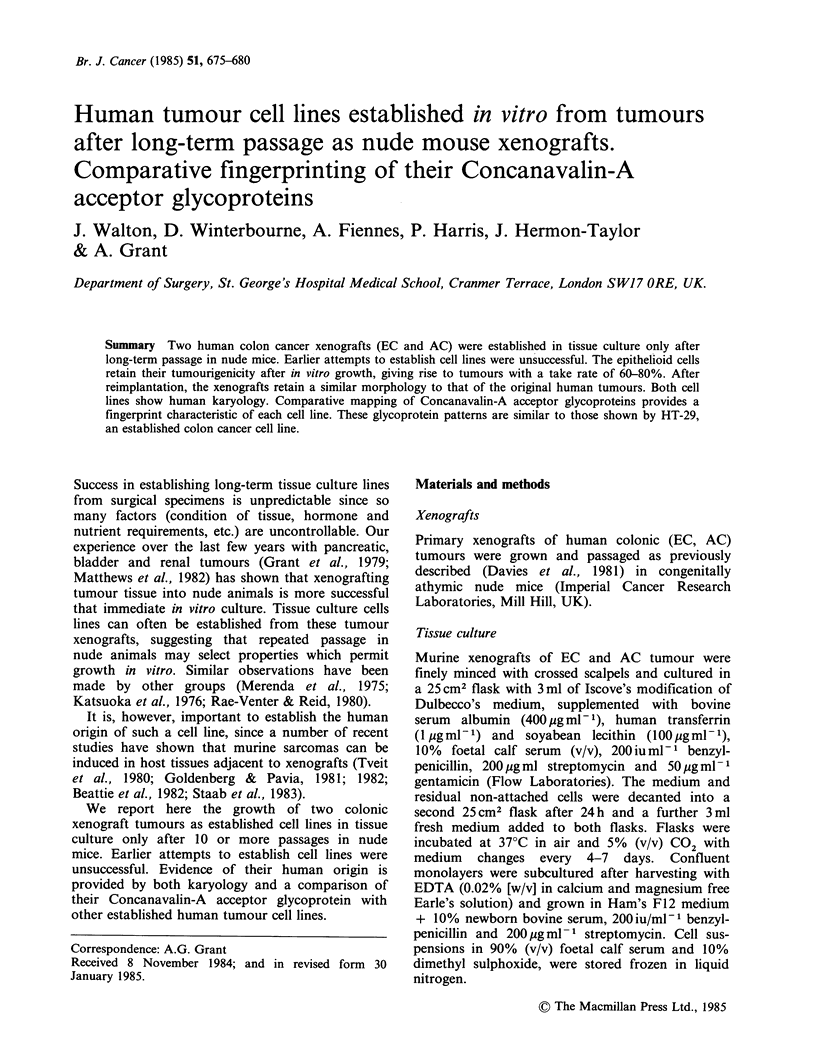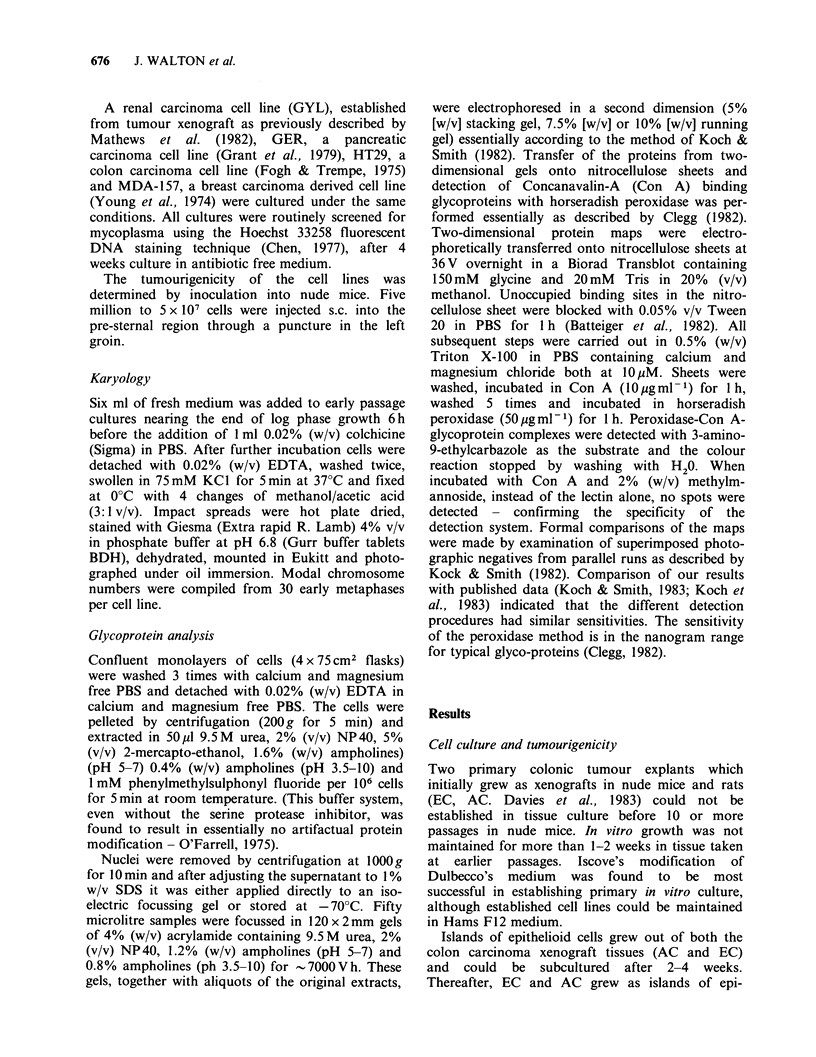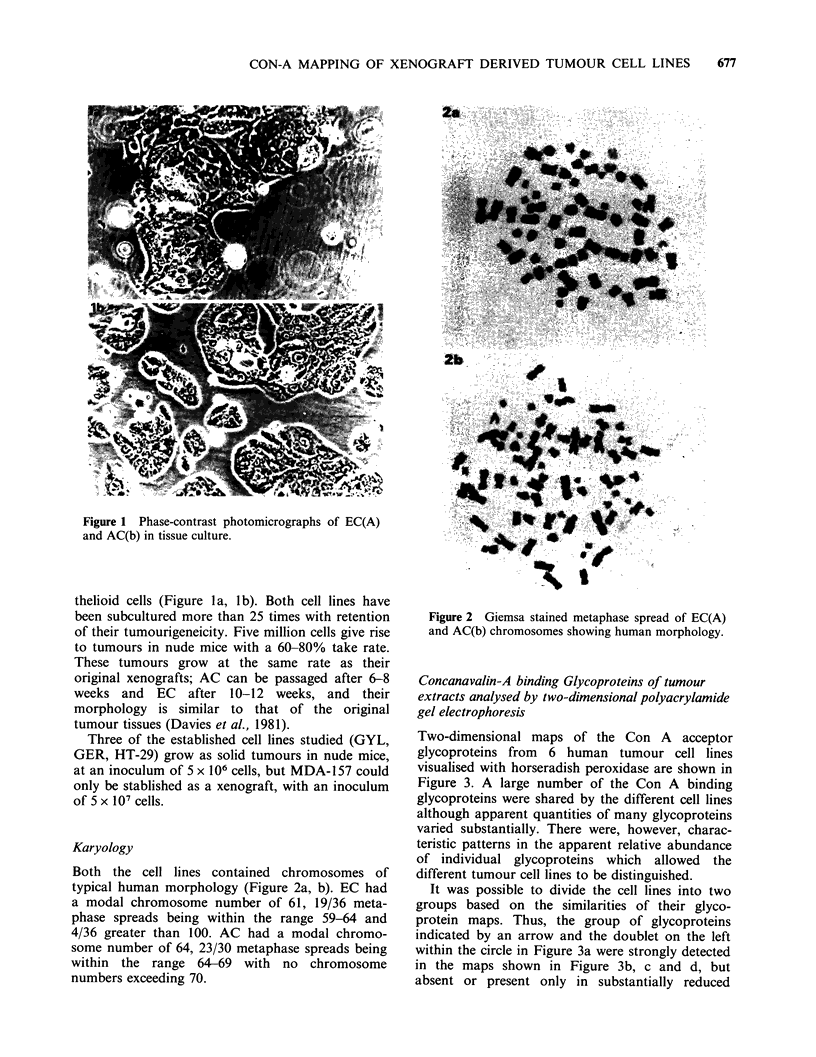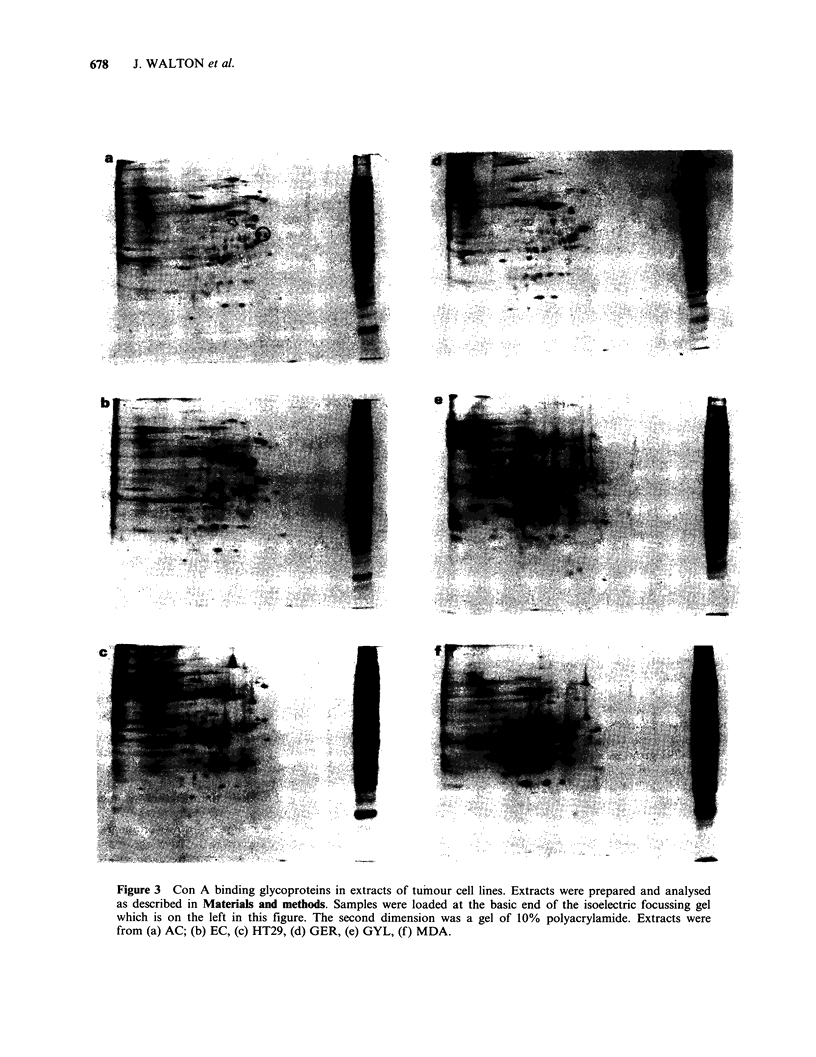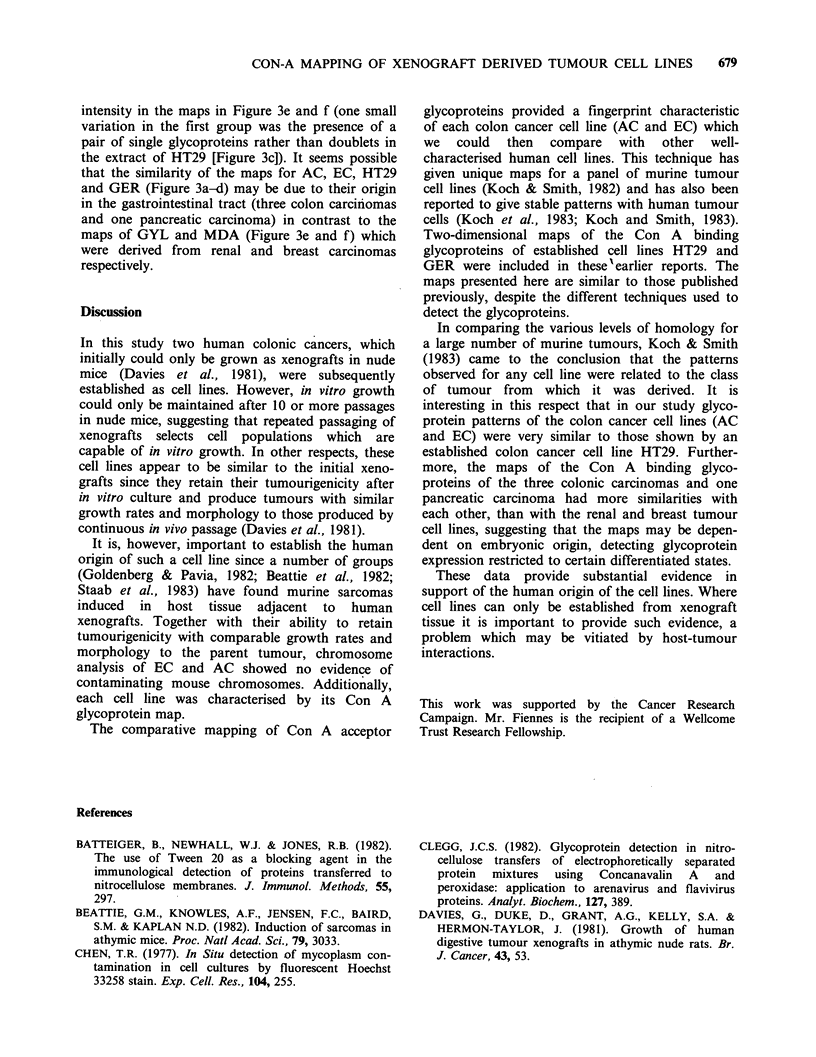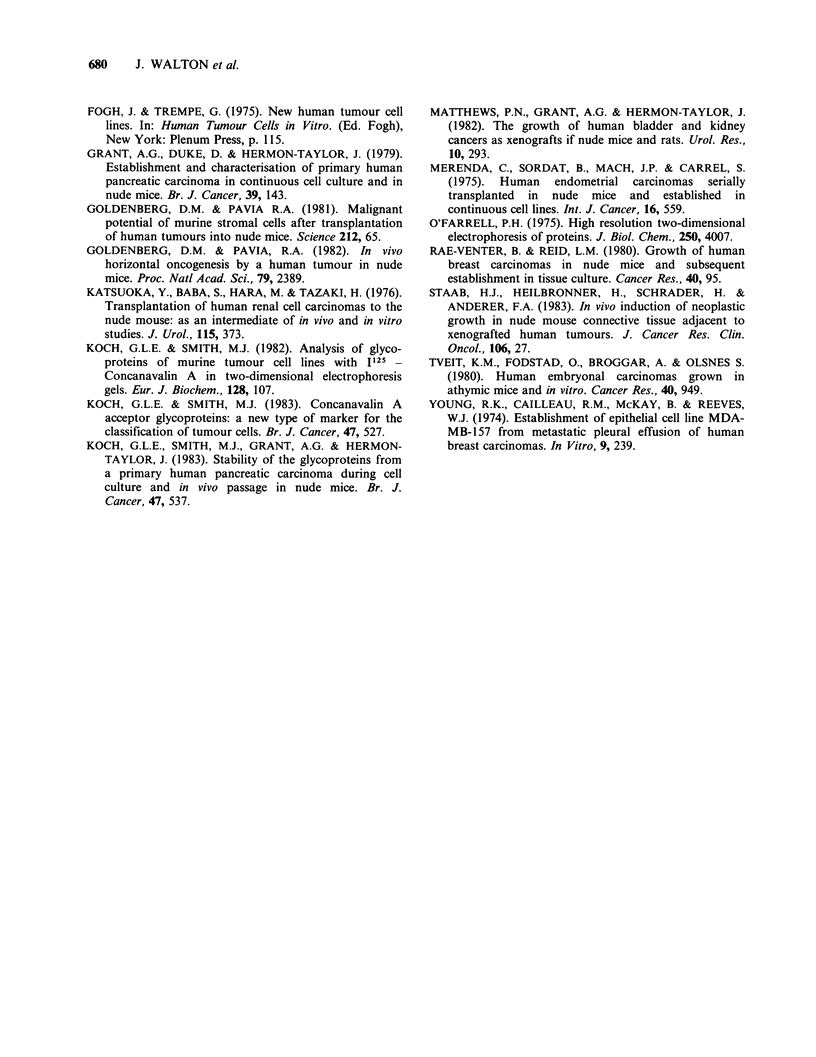# Human tumour cell lines established in vitro from tumours after long-term passage as nude mouse xenografts. Comparative fingerprinting of their concanavalin-A acceptor glycoproteins.

**DOI:** 10.1038/bjc.1985.102

**Published:** 1985-05

**Authors:** J. Walton, D. Winterbourne, A. Fiennes, P. Harris, J. Hermon-Taylor, A. Grant

## Abstract

**Images:**


*Br. J. Cancer* (1985) **51**, 675–680

### Human tumour cell lines established *in vitro* from tumours after long-term passage as nude mouse xenografts. Comparative fingerprinting of their Concanavalin-A acceptor glycoproteins

J. Walton, D. Winterbourne, A. Fiennes, P. Harris, J. Hermon-Taylor & A. Grant

*Department of Surgery, St. George's Hospital Medical School, Cranmer Terrace, London SW17 0RE, UK.*

**Summary** Two human colon cancer xenografts (EC and AC) were established in tissue culture only after long-term passage in nude mice. Earlier attempts to establish cell lines were unsuccessful. The epithelioid cells retain their tumourigenicity after *in vitro* growth, giving rise to tumours with a take rate of 60–80%. After reimplantation, the xenografts retain a similar morphology to that of the original human tumours. Both cell lines show human karyology. Comparative mapping of Concanavalin-A acceptor glycoproteins provides a fingerprint characteristic of each cell line. These glycoprotein patterns are similar to those shown by HT-29, an established colon cancer cell line.

Success in establishing long-term tissue culture lines from surgical specimens is unpredictable since so many factors (condition of tissue, hormone and nutrient requirements, etc.) are uncontrollable. Our experience over the last few years with pancreatic, bladder and renal tumours (Grant *et al.*, 1979; Matthews *et al.*, 1982) has shown that xenografting tumour tissue into nude animals is more successful that immediate *in vitro* culture. Tissue culture cells lines can often be established from these tumour xenografts, suggesting that repeated passage in nude animals may select properties which permit growth *in vitro*. Similar observations have been made by other groups (Merenda *et al.*, 1975; Katsuoka *et al.*, 1976; Rae-Venter & Reid, 1980).

It is, however, important to establish the human origin of such a cell line, since a number of recent studies have shown that murine sarcomas can be induced in host tissues adjacent to xenografts (Tveit *et al.*, 1980; Goldenberg & Pavia, 1981; 1982; Beattie *et al.*, 1982; Staab *et al.*, 1983).

We report here the growth of two colonic xenograft tumours as established cell lines in tissue culture only after 10 or more passages in nude mice. Earlier attempts to establish cell lines were unsuccessful. Evidence of their human origin is provided by both karyology and a comparison of their Concanavalin-A acceptor glycoprotein with other established human tumour cell lines.

Correspondence: A.G. Grant
Received 8 November 1984; and in revised form 30 January 1985.

### Materials and methods

### *Xenografts*

Primary xenografts of human colonic (EC, AC) tumours were grown and passaged as previously described (Davies *et al.*, 1981) in congenitally athymic nude mice (Imperial Cancer Research Laboratories, Mill Hill, UK).

### *Tissue culture*

Murine xenografts of EC and AC tumour were finely minced with crossed scalpels and cultured in a 25 $cm^2$ flask with 3 ml of Iscove's modification of Dulbecco's medium, supplemented with bovine serum albumin (400 $\mu g\,ml^{-1}$), human transferrin (1 $\mu g\,ml^{-1}$) and soyabean lecithin (100 $\mu g\,ml^{-1}$), 10% foetal calf serum (v/v), 200 iu $ml^{-1}$ benzylpenicillin, 200 $\mu g$ ml streptomycin and 50 $\mu g\,ml^{-1}$ gentamicin (Flow Laboratories). The medium and residual non-attached cells were decanted into a second 25 $cm^2$ flask after 24 h and a further 3 ml fresh medium added to both flasks. Flasks were incubated at 37°C in air and 5% (v/v) $CO_2$ with medium changes every 4–7 days. Confluent monolayers were subcultured after harvesting with EDTA (0.02% [w/v] in calcium and magnesium free Earle's solution) and grown in Ham's F12 medium + 10% newborn bovine serum, 200 iu/$ml^{-1}$ benzylpenicillin and 200 $\mu g\,ml^{-1}$ streptomycin. Cell suspensions in 90% (v/v) foetal calf serum and 10% dimethyl sulphoxide, were stored frozen in liquid nitrogen.

© The Macmillan Press Ltd., 1985

A renal carcinoma cell line (GYL), established from tumour xenograft as previously described by Mathews *et al.* (1982), GER, a pancreatic carcinoma cell line (Grant *et al.*, 1979), HT29, a colon carcinoma cell line (Fogh & Trempe, 1975) and MDA-157, a breast carcinoma derived cell line (Young *et al.*, 1974) were cultured under the same conditions. All cultures were routinely screened for mycoplasma using the Hoechst 33258 fluorescent DNA staining technique (Chen, 1977), after 4 weeks culture in antibiotic free medium.

The tumourigenicity of the cell lines was determined by inoculation into nude mice. Five million to $5 \times 10^7$ cells were injected s.c. into the pre-sternal region through a puncture in the left groin.

*Karyology*

Six ml of fresh medium was added to early passage cultures nearing the end of log phase growth 6 h before the addition of 1 ml 0.02% (w/v) colchicine (Sigma) in PBS. After further incubation cells were detached with 0.02% (w/v) EDTA, washed twice, swollen in 75 mM KC1 for 5 min at 37°C and fixed at 0°C with 4 changes of methanol/acetic acid (3:1 v/v). Impact spreads were hot plate dried, stained with Giesma (Extra rapid R. Lamb) 4% v/v in phosphate buffer at pH 6.8 (Gurr buffer tablets BDH), dehydrated, mounted in Eukitt and photographed under oil immersion. Modal chromosome numbers were compiled from 30 early metaphases per cell line.

*Glycoprotein analysis*

Confluent monolayers of cells ($4 \times 75\,cm^2$ flasks) were washed 3 times with calcium and magnesium free PBS and detached with 0.02% (w/v) EDTA in calcium and magnesium free PBS. The cells were pelleted by centrifugation (200 *g* for 5 min) and extracted in 50 $\mu$l 9.5 M urea, 2% (v/v) NP40, 5% (v/v) 2-mercapto-ethanol, 1.6% (w/v) ampholines) (pH 5–7) 0.4% (w/v) ampholines (pH 3.5–10) and 1 mM phenylmethylsulphonyl fluoride per $10^6$ cells for 5 min at room temperature. (This buffer system, even without the serine protease inhibitor, was found to result in essentially no artifactual protein modification – O'Farrell, 1975).

Nuclei were removed by centrifugation at 1000 *g* for 10 min and after adjusting the supernatant to 1% w/v SDS it was either applied directly to an iso-electric focussing gel or stored at −70°C. Fifty microlitre samples were focussed in 120 × 2 mm gels of 4% (w/v) acrylamide containing 9.5 M urea, 2% (v/v) NP40, 1.2% (w/v) ampholines (pH 5–7) and 0.8% ampholines (ph 3.5–10) for ~7000 V h. These gels, together with aliquots of the original extracts, were electrophoresed in a second dimension (5% [w/v] stacking gel, 7.5% [w/v] or 10% [w/v] running gel) essentially according to the method of Koch & Smith (1982). Transfer of the proteins from two-dimensional gels onto nitrocellulose sheets and detection of Concanavalin-A (Con A) binding glycoproteins with horseradish peroxidase was performed essentially as described by Clegg (1982). Two-dimensional protein maps were electrophoretically transferred onto nitrocellulose sheets at 36 V overnight in a Biorad Transblot containing 150 mM glycine and 20 mM Tris in 20% (v/v) methanol. Unoccupied binding sites in the nitrocellulose sheet were blocked with 0.05% v/v Tween 20 in PBS for 1 h (Batteiger *et al.*, 1982). All subsequent steps were carried out in 0.5% (w/v) Triton X-100 in PBS containing calcium and magnesium chloride both at 10 $\mu$M. Sheets were washed, incubated in Con A ($10\,\mu g\,ml^{-1}$) for 1 h, washed 5 times and incubated in horseradish peroxidase ($50\,\mu g\,ml^{-1}$) for 1 h. Peroxidase-Con A-glycoprotein complexes were detected with 3-amino-9-ethylcarbazole as the substrate and the colour reaction stopped by washing with $H_20$. When incubated with Con A and 2% (w/v) methylmannoside, instead of the lectin alone, no spots were detected – confirming the specificity of the detection system. Formal comparisons of the maps were made by examination of superimposed photographic negatives from parallel runs as described by Kock & Smith (1982). Comparison of our results with published data (Koch & Smith, 1983; Koch *et al.*, 1983) indicated that the different detection procedures had similar sensitivities. The sensitivity of the peroxidase method is in the nanogram range for typical glyco-proteins (Clegg, 1982).

### Results

*Cell culture and tumourigenicity*

Two primary colonic tumour explants which initially grew as xenografts in nude mice and rats (EC, AC. Davies *et al.*, 1983) could not be established in tissue culture before 10 or more passages in nude mice. *In vitro* growth was not maintained for more than 1–2 weeks in tissue taken at earlier passages. Iscove's modification of Dulbecco's medium was found to be most successful in establishing primary *in vitro* culture, although established cell lines could be maintained in Hams F12 medium.

Islands of epithelioid cells grew out of both the colon carcinoma xenograft tissues (AC and EC) and could be subcultured after 2–4 weeks. Thereafter, EC and AC grew as islands of epi-

**Figure 1** Phase-contrast photomicrographs of EC(A) and AC(b) in tissue culture.

thelioid cells (Figure 1a, 1b). Both cell lines have been subcultured more than 25 times with retention of their tumourigeneicity. Five million cells give rise to tumours in nude mice with a 60–80% take rate. These tumours grow at the same rate as their original xenografts; AC can be passaged after 6–8 weeks and EC after 10–12 weeks, and their morphology is similar to that of the original tumour tissues (Davies *et al.*, 1981).

Three of the established cell lines studied (GYL, GER, HT-29) grow as solid tumours in nude mice, at an inoculum of $5 \times 10^6$ cells, but MDA-157 could only be stablished as a xenograft, with an inoculum of $5 \times 10^7$ cells.

*Karyology*

Both the cell lines contained chromosomes of typical human morphology (Figure 2a, b). EC had a modal chromosome number of 61, 19/36 metaphase spreads being within the range 59–64 and 4/36 greater than 100. AC had a modal chromosome number of 64, 23/30 metaphase spreads being within the range 64–69 with no chromosome numbers exceeding 70.

**Figure 2** Giemsa stained metaphase spread of EC(A) and AC(b) chromosomes showing human morphology.

*Concanavalin-A binding Glycoproteins of tumour extracts analysed by two-dimensional polyacrylamide gel electrophoresis*

Two-dimensional maps of the Con A acceptor glycoproteins from 6 human tumour cell lines visualised with horseradish peroxidase are shown in Figure 3. A large number of the Con A binding glycoproteins were shared by the different cell lines although apparent quantities of many glycoproteins varied substantially. There were, however, characteristic patterns in the apparent relative abundance of individual glycoproteins which allowed the different tumour cell lines to be distinguished.

It was possible to divide the cell lines into two groups based on the similarities of their glycoprotein maps. Thus, the group of glycoproteins indicated by an arrow and the doublet on the left within the circle in Figure 3a were strongly detected in the maps shown in Figure 3b, c and d, but absent or present only in substantially reduced

**Figure 3** Con A binding glycoproteins in extracts of tumour cell lines. Extracts were prepared and analysed as described in **Materials and methods**. Samples were loaded at the basic end of the isoelectric focussing gel which is on the left in this figure. The second dimension was a gel of 10% polyacrylamide. Extracts were from (a) AC; (b) EC, (c) HT29, (d) GER, (e) GYL, (f) MDA.

intensity in the maps in Figure 3e and f (one small variation in the first group was the presence of a pair of single glycoproteins rather than doublets in the extract of HT29 [Figure 3c]). It seems possible that the similarity of the maps for AC, EC, HT29 and GER (Figure 3a–d) may be due to their origin in the gastrointestinal tract (three colon carcinomas and one pancreatic carcinoma) in contrast to the maps of GYL and MDA (Figure 3e and f) which were derived from renal and breast carcinomas respectively.

### Discussion

In this study two human colonic cancers, which initially could only be grown as xenografts in nude mice (Davies *et al.*, 1981), were subsequently established as cell lines. However, *in vitro* growth could only be maintained after 10 or more passages in nude mice, suggesting that repeated passaging of xenografts selects cell populations which are capable of *in vitro* growth. In other respects, these cell lines appear to be similar to the initial xenografts since they retain their tumourigenicity after *in vitro* culture and produce tumours with similar growth rates and morphology to those produced by continuous *in vivo* passage (Davies *et al.*, 1981).

It is, however, important to establish the human origin of such a cell line since a number of groups (Goldenberg & Pavia, 1982; Beattie *et al.*, 1982; Staab *et al.*, 1983) have found murine sarcomas induced in host tissue adjacent to human xenografts. Together with their ability to retain tumourigenicity with comparable growth rates and morphology to the parent tumour, chromosome analysis of EC and AC showed no evidence of contaminating mouse chromosomes. Additionally, each cell line was characterised by its Con A glycoprotein map.

The comparative mapping of Con A acceptor glycoproteins provided a fingerprint characteristic of each colon cancer cell line (AC and EC) which we could then compare with other well-characterised human cell lines. This technique has given unique maps for a panel of murine tumour cell lines (Koch & Smith, 1982) and has also been reported to give stable patterns with human tumour cells (Koch *et al.*, 1983; Koch and Smith, 1983). Two-dimensional maps of the Con A binding glycoproteins of established cell lines HT29 and GER were included in these earlier reports. The maps presented here are similar to those published previously, despite the different techniques used to detect the glycoproteins.

In comparing the various levels of homology for a large number of murine tumours, Koch & Smith (1983) came to the conclusion that the patterns observed for any cell line were related to the class of tumour from which it was derived. It is interesting in this respect that in our study glycoprotein patterns of the colon cancer cell lines (AC and EC) were very similar to those shown by an established colon cancer cell line HT29. Furthermore, the maps of the Con A binding glycoproteins of the three colonic carcinomas and one pancreatic carcinoma had more similarities with each other, than with the renal and breast tumour cell lines, suggesting that the maps may be dependent on embryonic origin, detecting glycoprotein expression restricted to certain differentiated states.

These data provide substantial evidence in support of the human origin of the cell lines. Where cell lines can only be established from xenograft tissue it is important to provide such evidence, a problem which may be vitiated by host-tumour interactions.

This work was supported by the Cancer Research Campaign. Mr. Fiennes is the recipient of a Wellcome Trust Research Fellowship.

### References

BATTEIGER, B., NEWHALL, W.J. & JONES, R.B. (1982). The use of Tween 20 as a blocking agent in the immunological detection of proteins transferred to nitrocellulose membranes. *J. Immunol. Methods*, **55**, 297.

BEATTIE, G.M., KNOWLES, A.F., JENSEN, F.C., BAIRD, S.M. & KAPLAN N.D. (1982). Induction of sarcomas in athymic mice. *Proc. Natl Acad. Sci.*, **79**, 3033.

CHEN, T.R. (1977). *In Situ* detection of mycoplasm contamination in cell cultures by fluorescent Hoechst 33258 stain. *Exp. Cell. Res.*, **104**, 255.

CLEGG, J.C.S. (1982). Glycoprotein detection in nitrocellulose transfers of electrophoretically separated protein mixtures using Concanavalin A and peroxidase: application to arenavirus and flavivirus proteins. *Analyt. Biochem.*, **127**, 389.

DAVIES, G., DUKE, D., GRANT, A.G., KELLY, S.A. & HERMON-TAYLOR, J. (1981). Growth of human digestive tumour xenografts in athymic nude rats. *Br. J. Cancer*, **43**, 53.

FOGH, J. & TREMPE, G. (1975). New human tumour cell lines. In: *Human Tumour Cells in Vitro*. (Ed. Fogh), New York: Plenum Press, p. 115.

GRANT, A.G., DUKE, D. & HERMON-TAYLOR, J. (1979). Establishment and characterisation of primary human pancreatic carcinoma in continuous cell culture and in nude mice. *Br. J. Cancer*, **39**, 143.

GOLDENBERG, D.M. & PAVIA R.A. (1981). Malignant potential of murine stromal cells after transplantation of human tumours into nude mice. *Science* **212**, 65.

GOLDENBERG, D.M. & PAVIA, R.A. (1982). *In vivo* horizontal oncogenesis by a human tumour in nude mice. *Proc. Natl Acad. Sci.*, **79**, 2389.

KATSUOKA, Y., BABA, S., HARA, M. & TAZAKI, H. (1976). Transplantation of human renal cell carcinomas to the nude mouse: as an intermediate of *in vivo* and *in vitro* studies. *J. Urol.*, **115**, 373.

KOCH, G.L.E. & SMITH, M.J. (1982). Analysis of glycoproteins of murine tumour cell lines with $I^{125}$ – Concanavalin A in two-dimensional electrophoresis gels. *Eur. J. Biochem.*, **128**, 107.

KOCH, G.L.E. & SMITH, M.J. (1983). Concanavalin A acceptor glycoproteins: a new type of marker for the classification of tumour cells. *Br. J. Cancer*, **47**, 527.

KOCH, G.L.E., SMITH, M.J., GRANT, A.G. & HERMON-TAYLOR, J. (1983). Stability of the glycoproteins from a primary human pancreatic carcinoma during cell culture and *in vivo* passage in nude mice. *Br. J. Cancer*, **47**, 537.

MATTHEWS, P.N., GRANT, A.G. & HERMON-TAYLOR, J. (1982). The growth of human bladder and kidney cancers as xenografts if nude mice and rats. *Urol. Res.*, **10**, 293.

MERENDA, C., SORDAT, B., MACH, J.P. & CARREL, S. (1975). Human endometrial carcinomas serially transplanted in nude mice and established in continuous cell lines. *Int. J. Cancer*, **16**, 559.

O'FARRELL, P.H. (1975). High resolution two-dimensional electrophoresis of proteins. *J. Biol. Chem.*, **250**, 4007.

RAE-VENTER, B. & REID, L.M. (1980). Growth of human breast carcinomas in nude mice and subsequent establishment in tissue culture. *Cancer Res.*, **40**, 95.

STAAB, H.J., HEILBRONNER, H., SCHRADER, H. & ANDERER, F.A. (1983). *In vivo* induction of neoplastic growth in nude mouse connective tissue adjacent to xenografted human tumours. *J. Cancer Res. Clin. Oncol.*, **106**, 27.

TVEIT, K.M., FODSTAD, O., BROGGAR, A. & OLSNES S. (1980). Human embryonal carcinomas grown in athymic mice and *in vitro*. *Cancer Res.*, **40**, 949.

YOUNG, R.K., CAILLEAU, R.M., McKAY, B. & REEVES, W.J. (1974). Establishment of epithelial cell line MDA-MB-157 from metastatic pleural effusion of human breast carcinomas. *In Vitro*, **9**, 239.